# Digital marketing techniques within online food retail platforms: a scoping review

**DOI:** 10.1186/s12916-025-04553-6

**Published:** 2025-12-10

**Authors:** Adyya Gupta, Catherine E. Huggins, Sara Salahshoornezhad, Rebecca Bennett, Kathryn Backholer, Anna Peeters

**Affiliations:** 1 Faculty of Health, School of Health and Social Development, Institute for Health Transformation, Global Centre for Preventive Health and Nutrition, Deakin University, Geelong, VIC Australia; 2 Victorian Health Promotion Foundation, Melbourne, VIC Australia

**Keywords:** Online food delivery, Online grocery services, Online food retail, Digital marketing techniques, Review

## Abstract

**Background:**

There is growing evidence that online food retail platforms (online food delivery, online grocery and meal kits) use a range of marketing techniques to promote unhealthy foods (energy-dense and nutrient-poor) to influence consumers’ food choices and food purchases. However, existing literature on this topic is dispersed (across public health and marketing disciplines) and has not been synthesised from a public health perspective. The aim of this review was to synthesise the evidence on the digital marketing techniques prevalent on online food retail platforms, what food products they promote and their potential influence on consumers’ online food purchase behaviours.

**Methods:**

A mix of health and business databases was searched for peer-reviewed papers published in the last ten years, to align with the time from when online food retail platforms grew in popularity. Included studies were mapped to a pre-defined list of digital marketing techniques, marketing mix strategies and across Cialdini’s principles of persuasion. All findings were described narratively.

**Results:**

A total of 3408 studies were screened, of which 16 studies were included in the review. Eleven studies examined online food delivery services, and five studies examined online grocery retail services. A range of well-established digital marketing techniques were identified, including algorithmic personalisation, push notifications, membership-based models, interactive tools such as sorting and filtering features, hyperlinks or prompts to create combo deals, site customisation offering personalised shopping experience, clickable banner advertisements and search engine optimisation. Whilst these are standard techniques in digital commerce, we found that they were often deployed to preferentially promote nutrient-poor foods. Specifically, seven studies highlighted the use of these marketing techniques to promote foods of poor nutritional quality, with marketing strategies reinforcing perceptions of value, tastiness and pleasure. Only two studies examined and reported a positive influence of using price promotion techniques on consumers’ unhealthy food purchase intention.

**Conclusions:**

Online food delivery and online grocery platforms use a range of digital marketing techniques to deliver content in highly persuasive ways, often promoting unhealthy food options. Future research should explore how these platforms can be leveraged to enable healthier population food choices and align with public health objectives.

**Supplementary Information:**

The online version contains supplementary material available at 10.1186/s12916-025-04553-6.

## Background

Diets high in sugar, salt and fats are among the leading risk factors for non-communicable diseases (NCDs) resulting in death and disability [1]. In the context of rapid digital transformation, a particular concern is the growth of online food retail services, driven by user-friendly platforms and the convenience of home delivery [2, 3]. Previous studies [4, 5] have characterised these services into three main types: (1) meal delivery applications (online food delivery (OFD) and other ready to eat meals), (2) online grocery platforms and (3) meal-kit subscription services. Emerging evidence suggests that young people, those residing in advantaged socio-economic areas and those with high educational qualification or incomes are common users of online food retail services [6, 7]. Whilst online food retail services vary in the type of food sold, they have been shown to predominantly promote foods with low nutritional value [8, 9]. This increases the likelihood of purchasing and over-consuming unhealthy food and beverages leading to adverse health impact.

The marketing of energy-dense, nutrient-poor foods and beverages plays a significant role in shaping dietary behaviours and food intake [10, 11]. Recent studies have demonstrated that in addition to conventional marketing practices (e.g. attractive food imagery, price discounts and meal deals), online food delivery and online grocery services use algorithmic techniques to track and predict individual preferences and deliver tailored marketing content to influence consumers’ food choices and purchases [9, 12]. Digital marketing refers to promotional activity delivered via digital media, that seeks to maximise impact through creative and/or analytical methods [13]. Some examples of the digital marketing techniques reported within the business literature [14, 15] includes:

- User Experience Strategy (UX): Creating interactive website designs through hyperlinks, search options, clickable banner advertisements to enhance usability and increase credibility
- Search engine marketing (SEM) (including search engine advertising (SEA) or optimisation (SEO)): to increase website visibility through organic ranking or paid search placement and guide the development of AI-driven personalisation algorithms
- Freemium to subscription-based marketing strategy: where businesses allow free use of a service for limited time and then charge a small recurring fee in exchange for access to premium, personalised services
- E-mail marketing: targeted email campaigns
- Mobile marketing: via SMS (short message service) or in-app push notifications for timely, personalised offers directly to consumers
- Pay-per-click advertising: a form of online advertising where advertisers pay a fee each time a user clicks on their ad, rather than paying for the ad to simply be displayed.
- Social media marketing: leveraging social media platforms and online communities to promote content, encourage reviews and stimulate users to generate content (UGC) and share their opinions and experiences through word-of-mouth online
- Affiliate marketing: involves partnering with other businesses or brands (affiliates) to promote products, share commission on sales and build brand reputation.
- Location based marketing: where a user’s location is used (e.g. using their phone’s Global Positioning System (GPS)) to deliver targeted promotional messages based on their geographical area
- Retargeting strategy: delivering targeted promotional messages to re-engage with users based on their previous interactions with the product or brands (e.g. visited a page, added specific items to the cart)

Despite evidence on the growing influence of these marketing practices [16], there is no public health evidence synthesising the key features of the digital marketing ecosystem that facilitates (unhealthy) food marketing in online food retail platforms. Furthermore, evidence on the impact of these digital marketing techniques on consumer food choices is limited. A clear understanding of how online food retail platforms use marketing techniques and how consumers interact with these marketing techniques is critical for developing evidence-based approaches to support healthier food environments. The existing literature on this topic is dispersed (across public health and marketing disciplines) and has not been synthesised to date from a public health perspective. Through a scoping review, we gathered relevant evidence from health and business literature to address the three aims outlined below:

A. To describe the digital marketing techniques used by online food retail platforms.
B. To describe the food products promoted by the digital marketing techniques.
C. To examine the influence of digital marketing techniques on consumer’s online food purchase behaviours.

## Methods

A systematic review protocol was developed a priori and registered in PROSPERO (The International prospective register of systematic reviews; registration number CRD42024536567) [17]. The process of developing the search strategy revealed the extensive breadth and complexity of the topic, and as a result, we revised our methodological approach and proceeded with a scoping review. From definition, a scoping review is conducted to examine the scope and nature of existing research on a broad topic and identify gaps in the existing research [18]. Thus, we applied a scoping review methodology as outlined by Arksey and O’Malley [19] to provide a comprehensive summary of the existing relevant literature, and therefore, did not perform a critical appraisal of the included studies. Importantly, all other elements outlined in the original protocol remain unchanged. For reporting of this scoping review, the PRISMA-ScR (Preferred Reporting Items for Systematic Reviews and Meta-Analyses extension for Scoping Reviews) guideline [20] was followed (Appendix A).

### Search strategy

A range of keywords and MeSH terms were chosen to capture studies that described any form of point-of-sale online food marketing techniques (e.g. push notification) and/or the creative content of the marketing message (e.g. use of price discount) used by online food retail platforms in the real world. Online food retail platforms include those where food can be ordered directly for pick-up or delivered directly from online grocery stores, independent restaurants or a third-party platform such as online food delivery services or offering ingredients for cook-at-home meals via meal kits. The search was conducted in a range of health and business databases, namely: Medline Complete, Web of Science, Business Source Complete, Communication and Mass Media Complete and ABI/Inform. We particularly selected Medline Complete and Web of Science for their comprehensive coverage of public health literature, including online food environment studies. Business Source Complete and ABI/Inform were included to capture literature on retail, marketing and commercial aspects of online food environments. Communication and Mass Media Complete was selected to identify studies related to the communication of online food marketing and messaging to promote sales of food on online food platforms. Database search terms were categorised under the following hedge terms: online food retail types and online food marketing techniques (Appendix B). The keywords were initially informed by prior literature [4, 15, 21, 22] and refined through iterative pilot searches to ensure relevance and coverage across both public health and business domains. A pilot search was conducted to identify commonly used terminology and adjust for variations across disciplines. For example, the term ‘e-commerce’ was specifically added to the search strategy to capture relevant studies in business literature. The search strategy was reviewed internally by team members with expertise in systematic and scoping review methodologies. A forward search (citation search of included papers) and backward search (reference lists of all relevant articles) were performed to capture any potentially relevant peer-reviewed published literature. No grey literature such as book chapters, conference abstracts, editorials, opinion pieces, reports, commentaries, dissertations and policy documents were included. All peer-reviewed journal articles (English language only) that were published in the last 10 years (2015 to December 2024) were considered to align with the time from when online food retail platforms grew in popularity and to identify the most recent literature on the topic. These were subjected to selection criteria as described below.

### Study selection

A PCC (population, concept and context) criterion was developed to inform the study selection (Table 1). Articles were eligible for inclusion if they described any form of point-of-sale online food marketing techniques (e.g. push notification) and/or the creative content of the marketing message (e.g. use of price discount or appealing food images) used by online food retail platforms in the real world to promote (healthy and unhealthy) foods and non-alcoholic beverages. Online food marketing refers to promotional activities implemented by and directly on online food retail platforms in real-world settings to influence consumer food and beverage choices. These techniques include, but are not limited to, push notifications, banner advertisements, content marketing, loyalty programmes and personalised recommendations. Studies that described marketing techniques to promote alcoholic beverages were excluded. Studies that examined marketing techniques on other online settings such as social media platforms (e.g. Facebook), non-grocery e-commerce sites (e.g. clothing retailers), brand sites (e.g. Coca-Cola, Pepsi), and content streaming sites (e.g. YouTube) and physical food retail stores, television, or postal mail were excluded. All study designs were eligible for inclusion in the review where studies were conducted in the real world to capture the multitude of actual marketing techniques applied on online food retail platforms. This approach helped to understand how these techniques are being utilised in the real world to promote food, how and what consumers are being exposed to in real time and their influence on actual consumer behaviour. Drawing insights on real-time marketing tactics from the business literature enabled us to meaningfully strengthen the foundation for informed and responsive public health policy development. Experimental studies with discrete choice designs conducted in simulated online food retail settings with a hypothetical scenario of online food marketing techniques to predict individuals’ food choices were excluded.

**Table 1:** Study Selection Criteria

Criteria	Inclusion	Exclusion
Population	People from all countries, ages and genders	N/A
Concept	Studies that describe any form of point-of-sale online food marketing techniques (e.g. push notification) and/or the creative content of the marketing message (e.g. use of price discount) used by online food retail platforms in the real world to promote (healthy and unhealthy) foods and non-alcoholic beverages	Studies that describe marketing techniques to promote alcoholic beverages will be excluded
Context	Studies conducted on online food retail platforms in the real world. Online food retail platforms include those where food can be ordered directly for pick-up or delivered directly from online grocery stores, independent restaurants or from other online food retailers or indirectly from a third-party platform	Other online settings such as social media platforms (e.g. Facebook), non-grocery e-commerce sites (e.g. clothing retailers), brand sites (e.g. Coca-Cola, Pepsi), online school canteens and content streaming sites (e.g. YouTube); physical food retail stores; television; postal mail Influencer marketing, whilst often delivered through online channels such as social media, typically operates outside the direct control of online food retail platforms and will be excluded, unless explicitly integrated into the platform’s own marketing strategy
Study Design	Peer-reviewed journal articles including Quantitative (quasi-experimental, experimental, longitudinal, cross-sectional, observational) and qualitative studies will be included	Commentaries, conference abstracts, editorials, laboratory-based or modelling studies Experimental studies with discrete choice designs conducted in a simulated online food retail setting with a hypothetical scenario of online food marketing techniques to predict individuals’ food choice
Others	All studies published in the English language from 2015 to August 2024	Grey literature such as book chapters, conference abstracts, editorials, opinion pieces, reports, commentaries, dissertations and policy documents

All studies identified from the database search were exported to Covidence—a web-based software that allows a streamlined process for conducting systematic and other literature reviews [23]. After duplicate articles were removed, titles, abstracts and full texts were screened by three authors independently (AG, SS and RB) against the inclusion and exclusion criteria. Any discrepancies were resolved via consensus between the authors.

### Data extraction

A pilot data extraction template was developed, and data extraction of four potentially included studies was undertaken by the primary author and was reviewed by two other authors (KB and CEH). The accuracy of the information extracted was discussed among the authors, and the final data were extracted from all included articles by the primary author. Data were collated into a pre-determined matrix that included publication details (author-year, study design and country), type of journal (health or business), type of online food retail platform (e.g. online grocery stores, independent restaurants or online food delivery services), description of the digital marketing technique/s reported (e.g. push notification), the creative content of the marketing message reported (e.g. use of price discount or appealing food images), type of food promoted and any potential impact of online food retail marketing techniques on consumer purchase (food purchases; knowledge, attitudes, preferences towards food selection/choice).

### Data analysis

A narrative synthesis of the data was undertaken to address the research aims. To address research aim A, the digital marketing techniques reported in the included studies were identified and mapped to the pre-defined list of ten most commonly applied digital marketing techniques as described in the background [14, 15] (namely, UX, SEM (including SEA or SEO), Freemium to subscription-based marketing, E-mail marketing, mobile marketing, pay-per-click advertising, social media marketing, affiliate marketing, location-based marketing and retargeting strategy). The creative content of the marketing messages, which included elements designed to be attractive or engaging to the user, was then identified and mapped according to the 4P’s of the marketing mix strategies (e.g. price, promotions) [24] and open coded to capture additional marketing strategies such as nudges. Both the marketing techniques and marketing messages were compared based on the type of platform, i.e. online grocery services vs. online food delivery services. We further examined the marketing techniques across Cialdini’s six principles of persuasion [25, 26] to assess the persuasiveness of the marketing techniques (and strategies) to inform purchasing and consumption decisions. These include Reciprocity (offering something of value to enhance user engagement such as free delivery), Commitment (beliefs consistent with values for sustained actions and continued use such as consumer satisfaction, consumer reviews), Social Proof (validation based on what others are doing such as popularity cues), Liking (physical attractiveness, enhancing hedonic experiences such as appealing food images), Authority (trust, reputation such as brand reputation, consumer loyalty), Scarcity (short supply or short-term supply such as price promotions) (see Appendix C for codebook).

To address research aim B, we described the evidence on the food item promoted and compared it based on the type of online food retail platform. We then determined the healthiness of the food item based on Australian Dietary Guidelines [27] that categorise the food item as per their nutritional value, i.e. healthy food included nutrient-rich, low in unhealthy fats and added sugars and minimally processed, for example, fruits, vegetables, whole grains, lean protein and dairy. Discretionary foods are high in saturated fat, added sugar and sodium, for example, fried foods, chocolate, crisps, cakes, biscuits. This was only done for studies that did not assess the healthiness of the food based on the country-specific guidelines.

Finally, to address research aim C, each study was examined for the digital marketing techniques, strategies assessed, their underpinning principle of persuasion and outcome measures reported. We also extracted details on the direction and magnitude of the influence of online food marketing techniques on consumers’ food purchase behaviour-related outcomes, where reported, to infer the findings. We examined our findings based on the type of platform to identify common patterns and contextual differences (across age groups, gender and other socio-demographic characteristics).

## Results

### Search results

**Fig. 1:**
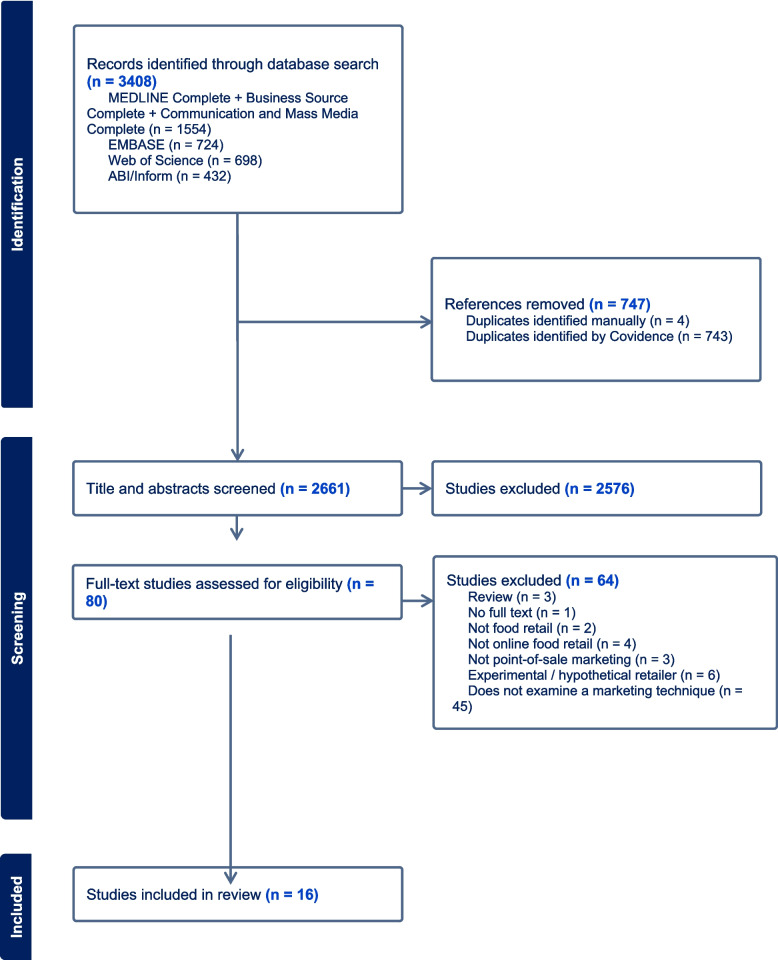
PRISMA flowchart

A total of 3408 published peer-reviewed articles were identified from database searches. Following the removal of duplicates (*n* = 747), title and abstract screening was conducted for 2661 articles. Of these, 80 full-text articles were assessed for their eligibility, and 64 articles were excluded based on reasons listed in the PRISMA flowchart (Fig. 1: PRISMA). A total of 16 unique articles were considered eligible for inclusion in this review.

### Overview of the included studies (Table 2)

The 16 included studies [9, 12, 28–41] were published between 2016 and 2024. Nine [9, 12, 28–30, 32, 34, 36, 39] were published in health journals and seven [31, 33, 35, 37, 38, 40, 41] were published in business journals. Of the nine studies published in health journals, eight were cross-sectional, and one was a cohort study [34]. All seven studies published in business journals were cross-sectional studies [31, 33, 35, 37, 38, 40, 41].

Eleven studies examined online food delivery services, and the remaining five studies examined online grocery retail services. No studies examining marketing techniques on meal kit platforms were identified. Seven studies were conducted in high-income countries, including two each in Australia and the United States, and one each in New Zealand, Portugal and Finland. Seven were conducted in upper middle-income countries, including three in Brazil, two in Thailand and one each in Indonesia and China, and two studies published in business journals were conducted in a low-income country, India.

The sample size of the participants reported in six studies ranged from 30 to 1049. A small number of studies described the demographic characteristics (age and gender) of the participants. Over half of the participants in included studies [31, 33, 35, 36, 40, 41] were females and between 18 and 67 years of age. Only two studies assessed outcomes related to consumers’ online food purchasing intentions [33, 41] (Table 2).

**Table 2:** Study characteristics (*n* = 16 studies)

Year	Publication details (author, year, study design and country and study title)	Type of journal (health or business)	Aim of the study	Online food marketing technique by platform type	Creative content of the marketing message (using 4Ps and beyond)	Food item(s) promoted	Potential impact of online food marketing on consumer food purchase-related outcomes
Online food delivery services (*n* = 11)							
2020	Horta PM., et al. 2020 [28]; Cross-sectional study (quantitative); Brazil Digital food environment of a Brazilian metropolis: food availability and marketing strategies used by delivery apps	Health	To examine food availability and the use of marketing strategies by two food delivery apps in a Brazilian metropolis	Not reported	Price discounts and use of photos	Ultra-processed beverages; ice cream and candies; sandwiches; fried savoury snacks and pizzas	Not assessed
2020	Horta PM., et al. 2020 [12]; Cross-sectional study (quantitative); Brazil Digital food environment during the coronavirus disease 2019 (COVID-19) pandemic in Brazil: an analysis of food advertising in an online food delivery platform	Health	To describe the advertisements published in an OFD platform in Brazilian capitals, during the 13th and 14th weeks of the pandemic	Not reported	Free delivery, discounts, use of photos, combos (a combination of food items and/or drinks offered at a discount) and messages of healthiness, economy, tastiness and pleasure	*Free delivery* on ice cream, candies or salty packages snacks and pizza. *Combo deals* included natural juices or smoothies, ultra-processed beverage, sandwiches and pizzas. *Messages of healthiness* appeared on natural juices or smoothies; vegetables; and traditional meals (dishes predominantly made with unprocessed and minimally processed foods very typical in Brazil) and pasta. *Economy messages* appeared on traditional meals or pasta. *Tasty and pleasure messages* were more present on sandwiches and savoury snacks	Not assessed
2021	Wang C., et al. 2021 [29]; Cross-sectional study (quantitative); Australia Hunger for Home Delivery: Cross-Sectional Analysis of the Nutritional Quality of Complete Menus on an Online Food Delivery Platform in Australia	Health	To evaluate the nutritional quality and marketing attributes of offerings from independent takeaway outlets available on Sydney’s market-leading OFD platform (UberEats)	Not reported	Popularity cues (category of ‘Most Popular’), price, value bundles, use of image, nutritional information and dietary labelling	Discretionary food and beverages made up the majority of menus and were more likely to be *most popular*, accompanied by an *image* and offered as a *value bundle* (including catering and party packs, meal deals and family deals) than Five Food Group (FFG) menu items	Not assessed
2021	Jia SS., et al. 2021 [30]; Cross-sectional study (quantitative), Australia #SupportLocal: how online food delivery services leveraged the COVID-19 pandemic to promote food and beverages on Instagram	Health	To explore the promotion of discretionary foods/beverages and marketing strategies employed by the top three online food delivery services' (OFDS) Instagram accounts in three countries before and during the coronavirus disease 2019 (COVID-19) pandemic	Links to additional content, external pages and sponsorships or partnerships (events that the brand supports or brands/service partners)	Use of photos, health claims **COVID-19 specific marketing strategies**: Use of hashtags (OpenforDelivery or #ThankADasher), or post about ‘staying in’ or ‘quarantine entertainment’, combatting the pandemic, selling social distancing, messages of showing support for frontline workers and increasing virtual interaction	Discretionary food and beverages made up the majority of menus and were more likely to be targeted with marketing strategies	Not assessed
2022	Jitsoonthornchaikul M. 2022 [31]; Cross-sectional study (mixed methods); Bangkok, Thailand An empirical study on the service marketing factors influencing the need of consumers for an online food ordering delivery using subscription-based model	Business	To empirically explore several service marketing factors influencing the needs of consumers regarding online food ordering and delivery services via use of a subscription-based model	Subscription-based business model	Product quality, price discount, place (convenience, suitability and approachability of the delivery service), promotion (advertising, public relations, sales), people (delivery person), purchasing process and physical evidence (includes the infrastructure, the digital platform, advanced technology, information systems, networking and website design)	Not reported	Not assessed
2022	Mahawar N., et al. 2022 [32]; Cross-sectional study (quantitative); New Zealand Unhealthy Food at Your Fingertips: Cross-Sectional Analysis of the Nutritional Quality of Restaurants and Takeaway Outlets on an Online Food Delivery Platform in New Zealand	Health	To examine the nutritional quality and marketing attributes of menu items from popular independent and franchise restaurants and takeaway outlets on New Zealand’s market-leading OFD platform (UberEATS)	Not reported	Popularity cue (Uber eats category of ‘Most Popular’ and ‘Picked-for-you’), price, photo of menu item, value bundles (meal deals and family deals), special promotions (price-based promotions- ‘Buy 1, get 1 free’, Free with $20 purchase (add to cart), nutritional information and dietary labelling	Discretionary food and beverages made up the majority of the menus and, compared to FFG menu items, were more likely to be in the popularised section, accompanied by a photo, offered as a value bundle and included in special promotions	Not assessed
2022	Sari PN., et al. 2022 [33]; Cross-sectional study (quantitative); Indonesia Buying behaviour in online food delivery applications during the Covid-19 pandemic	Business	To examine the effect of discount framing, brand reputation, purchase intention and actual behaviour based on online food delivery applications during the COVID-19 pandemic	Brand perception (via good service quality and product quality)	Discount framing (as absolute saving or percentage discount)	Not reported	Brand perception (via good service quality and product quality) and price-centric promotions (via discount framing) had a positive effect on purchase intention (measured via intention-related statements)
2022	Horta MP., et al. 2022 [34]; Cohort Study, Brazil Food promoted on an online food delivery platform in a Brazilian metropolis during the coronavirus disease (COVID-19) pandemic: a longitudinal analysis	Health	To analyse food advertised on an online food delivery (OFD) platform during 16 weeks of the COVID-19 pandemic in Brazil	Not reported	Photos, discounts, ‘combo deals’ and messages on healthiness, value for the money, tastiness and pleasure	Natural juices and smoothies; vegetables; fruits; traditional meals and pasta; ultra-processed beverages; ice cream, candies and salty packaged snacks; sandwiches; savoury snacks; and pizza	Not assessed
2022	Rita P., et al. 2022 [35]; Cross-sectional study (mixed methods); Portugal The role of information for the customer journey in mobile food ordering apps	Business	To determine how to influence the customer journey of mobile food ordering applications (MFOAs) users	Web personalisation strategies (content personalisation–recommendations and offers), functional personalisation (select favourite restaurants and meals), and system-driven personalisation (ability to select and deselect restaurants)	Firm-generated information (such as ads) and user-generated information (word-of-mouth, customer reviews)	Not reported	Not assessed
2023	Pandey A. and Wang, JX. 2023 [36], cross-sectional study (quantitative); Thailand The Impact of Marketing Mix (4Ps) on Brand Equity towards Customer Loyalty: A Case Study of the Food Delivery Industry in Thailand	Health	To examine the relationship between the marketing mix (4Ps) and brand equity in the food delivery industry in Thailand	Brand equity (reputation brand awareness)	Price; Product type; Placement of the product; Promotion	Not reported	Not assessed
2023	Anil A., et al. 2023 [37]; Cross-sectional study (quantitative); India Effect of push-up notifications by online food delivery apps on customer behaviour in Chennai	Business	To investigate the impact of promotional mobile direct marketing campaigns sent via push-up notifications from online food delivery apps on purchase behaviour in Chennai, India	Mobile direct marketing campaign (Push-up notifications)	Promotions	Not reported	Not assessed
Online grocery services (*n* = 5)							
2016	Banerjee T. and Banerjee A. 2016 [38]; cross-sectional study (mixed methods); India Web Content Analysis of Online Grocery Shopping Web Sites in India	Business journal	To evaluate online grocery shopping web sites catering to customers primarily in India	Parameters used by Search engine optimisation (SEO)—daily visitors, daily page views, Alexa rank, page speed score, images on the site in % compared to other content on the site, traffic source on the website of which organic search traffic in %, web site worth in dollars, income per day in dollars	User-generated information (customer reviews on timely delivery)	Not reported	Not assessed
2021	Headrick G., et al. 2021 [39]; Cross-sectional study (quantitative); United States Content Analysis of Online Grocery Retail Policies and Practices Affecting Healthy Food Access	Health journal	To describe the policies and practices of online grocery retailers that may affect healthy food access, including retailers participating in the US Department of Agriculture's Supplemental Nutrition Assistance Program Online Purchasing Pilot	Personalisation of offers and advertisements (targeting ads, coupons, or promotions)	Price; Product type; Placement of the product; Promotion, nutritional information and dietary labelling	Not reported	Not assessed
2022	Hallikainen H., et al., 2022 [40]; Cross-sectional study (quantitative); Finland Consequences of personalised product recommendations and price promotions in online grocery shopping	Business journal	To assess how personalised product recommendations (recommendation agents) and price promotions (algorithmic pricing) compensate for the negative impact that consumer's perceived cognitive effort causes on loyalty	Web personalisation approaches (recommendation agents and algorithmic pricing)	Product recommendation, price promotions	Not reported	Not assessed
2022	Moran AJ., et al. 2022 [9]; Cross-sectional study (quantitative); USA Food Marketing Practices of Major Online Grocery Retailers in the United States, 2019–2020	Health Journal	To develop and apply a coding instrument to describe food marketing and the nutritional quality of marketed products in online grocery stores	Website customisation for personalised storefronts (sort and filter features that allow customers to view products according to certain attributes), site navigation (e.g. features that allow customers to customise the look and feel of the site), and shopping tools (e.g. shopping lists) Brand reputation (reduced membership fees for SNAP participants), Sponsorship (disclosure of product)	Product mix (i.e. assortment of products that can be viewed on the site); price (discounts, rewards and time-limited details (e.g. a weekly flyer)); placement (production recommendations, the order in which search results are presented on the site, product advertisements and other branded site content); and promotion (includes user feedback (ratings and reviews), product images, recipes, social media interaction and point-of-purchase information (e.g. food labelling))	64% of marketed foods and beverages were ultra processed. Of the ultra-processed foods, 69% were excessive in sodium, 49% were excessive in free sugars, 40% were excessive in saturated fats, and 4% contained low- or no-calorie sweeteners. The top five marketed food and beverage categories reported were: candy, sweets and snacks (17.3%); fruit, vegetables and legumes (16.7%); bread, cereal and grains (10.8%); milk, yogurt and cheese (9.7%); and sugary drinks (6.9%)	Not assessed
2023	Zhao Y., et al. 2023 [41]; Cross-sectional study (mixed-methods); China How brick-and-mortar retailers and grocery delivery platforms (GDP) influence purchase intention?	Business journal	To explore the role of brick-and-mortar (B&M) retailers and GDPs in online grocery shopping (OGS) experience, attitude and continuous purchase intention under the platform model of online grocery retailing	Not reported	Price; Promotion, Product type; Placement of the product; good customer service and instant delivery	Not reported	Good customer service, price value and instant delivery influence a positive attitude towards continuous purchase intentions (measured using a survey) using GDP

Below we present the results highlighting key patterns across studies, analysed by type of online food retail platform addressing each of the research aims (Appendix C).

#### Online food marketing technique/s and creative content of the marketing message

**Fig. 2:**
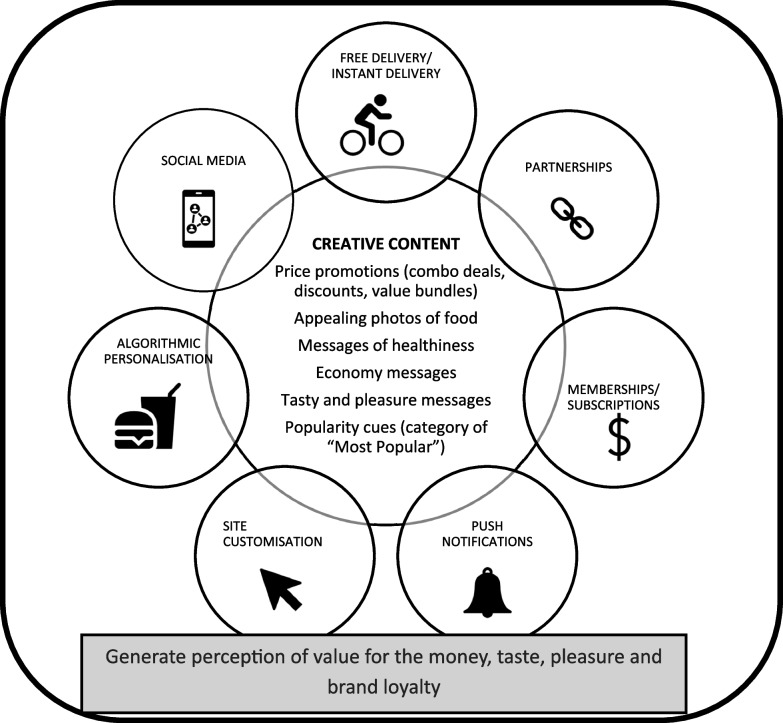
Digital marketing strategies on online food retail platforms

Ten (six on online food delivery services and four on online grocery services) of the sixteen studies described some form of digital marketing techniques, and all studies described a range of creative contents of the marketing messages (Fig. 2). Affiliate marketing (*n* = 6 studies) was the most utilised technique, both as standalone and in combination with other techniques such as social media marketing, subscription-based marketing and personalisation strategies on both online food delivery and online grocery services. This was followed by web customisation techniques (*n* = 3) and the push notification marketing technique (*n* = 1). A range of marketing mix strategies was utilised to inform the creative content of marketing messages. Over half of the studies (*n* = 14) used a variety of price promotion appeals ranging from combo deals to price discounts, followed by appealing food images (*n* = 7), popularity cues (*n* = 5), health claims among others. Upon examining these marketing techniques against Cialdini’s six principles of persuasion, twelve of the sixteen studies were found to utilise a combination of three principles, namely, ‘scarcity’, ‘liking’ and ‘social proofing’, emphasising value for the money, taste and pleasure, alongside building brand reputation.

#### Online food delivery services

Six out of 11 studies of OFD services described five types of digital marketing techniques used, namely affiliate marketing technique (*n* = 2 studies), affiliate marketing technique combined with social media marketing (*n* = 1), mobile marketing (*n* = 1), subscription-based marketing technique (*n* = 1) and search engine marketing technique to deploy AI-driven personalisation algorithms (*n* = 1). All reported on the various forms of the creative content of the marketing messages. Whilst the most reported across all studies was the price promotions of the traditional 4Ps of the marketing mix strategies to create a sense of urgency about specific food items (‘scarcity’), one study reported an additional 3Ps (including quality of service delivery (people), ordering to receiving delivery (process) and tracking orders (physical evidence)) to build trust in the service (‘Authority’) used by online food delivery services.

Two cross-sectional studies, conducted in Indonesia [33] and Thailand [36], described the use of affiliate marketing techniques involving partnering with brands (affiliates) to leverage one or more of the 4Ps. For example, framing price discounts (absolute saving that increases the initial selling price and then crosses out the price to be replaced with a new lower price or percentage discount claims), offering good product quality, and the strategic placement and promotion of the products, in partnerships with multiple restaurants to build brand reputation. One [30] of the three [29, 30, 32] cross-sectional studies described how three leading online food delivery services in Australia, the UK and the USA, during COVID-19, combined social media marketing techniques with affiliate marketing techniques to promote their brand using appealing photos of food and health claims. For example, they used hashtags such as #ThanksADasher or #OpenforDelivery and included posts about ‘staying in’ or ‘quarantine entertainment’, combatting the pandemic, selling social distancing and including messages of showing support for frontline workers, suggesting attempts to build brand awareness, support and reputation. Whilst the other two studies [29, 32] described other marketing strategies such as the use of popularity cues (category of ‘Most Popular’), price promotions (value bundles), the use of image, nutritional information and dietary labelling prevalent on independent takeaway outlets available on online food delivery services operating in Australia and New Zealand. Creative messages of healthiness (promoting traditional meals typical in Brazil) along with economy messages (indicating price promotions) combined with messages indicating taste and pleasure (appealing photos) were reported to be used by two leading online food delivery service apps in three studies conducted in Brazil, prior to [28] and during COVID-19 [12, 34]. One cross-sectional study conducted in India described push-up notifications as a marketing technique applied on a locally designed online food delivery app to alert users of promotional campaigns [37]. One cross-sectional mixed methods study conducted in Bangkok, Thailand, described how the 7Ps of marketing mix (product quality, price, place (other digital social media platforms), promotions (advertising, sales promotions, trade promotions and direct promotions), people (service delivery quality), process (ordering process, confirmation process, timely tracking and the delivery process), and physical evidence (the digital platform, advanced technology, information system to track orders) influenced consumers to subscribe to online food delivery services via a subscription-based marketing model. The study found that of the seven marketing strategies, four strategies including product quality, promotions, process and physical evidence influenced 1097 Thai consumers to be members of an online food ordering business subscription [31]. The study described the use of subscription-based marketing techniques as a tool for consumer acquisition, retention, promotion (upselling and cross-selling), and forecasting consumer demands based on consumer data. A range of algorithmic personalisation strategies including content personalisation (tailored recommendations and offers), functional personalisation (selection of favourite restaurants and meals), and system-driven personalisation (ability to select and deselect restaurants) were applied to promote advertisements and user-generated content (word-of-mouth, customer reviews) and generate consumer satisfaction and intention to use online food delivery services in a study conducted in Portugal [35].

#### Online grocery services

Four out of five studies examining online grocery services described four types of digital marketing techniques used either as standalone or in combination with each other. This included search engine marketing techniques to deploy AI-driven personalisation algorithms and a combination of website customisation, subscription-based marketing strategies and affiliate marketing techniques. All five reported on utilising the 4Ps of the marketing mix strategies and leveraged the principles of ‘scarcity’, ‘liking’ and ‘social proofing’.

Two studies [9, 39], conducted in the USA, analysed 21 online grocery retail websites between 2019 and 2020, described the use of multiple marketing techniques including website customisation, a membership-based model (reduced membership fees for Supplemental Nutrition Assistance Program (SNAP) participants) and an affiliate marketing model partnering with other sponsors (where they collected personal information from shoppers and automatically shared data with affiliated companies) to apply multiple marketing messages on the platform. The marketing messages reported include product mix (i.e. assortment of products that can be viewed on the site); price promotions (discounts, rewards and time-limited details (e.g. a weekly flyer)); placement strategies (production recommendations, the order in which search results are presented on the site, product advertisements and other branded site content); and promotion (includes user feedback (ratings and reviews), product images, recipes, social media interaction and point-of-purchase information (e.g. food labelling)). The 4Ps, along with good customer service and instant delivery influence, were also reported as key marketing strategies on an online grocery service in China [41]. Personalised product recommendations (recommendation agents) and price promotions (algorithmic pricing) were reported to be used by one of the largest grocery chains in Finland that covers over 65% of Finnish households [40]. A cross-sectional study analysed the web content of 10 leading Indian online grocery platforms and reported the use of SEO marketing techniques whereby e-retailers capture consumer preferences to tailor their websites to increase the desirability of products and subsequently enhance the visibility of their online grocery retail business [38].

#### The food item promoted and its healthiness

Less than half of the studies (*n* = 7) reported what kind of food item(s) were promoted in marketing on online food delivery services (*n* = 6) and online grocery service (*n* = 1). The food items most promoted were classified as discretionary food and beverages by the studies themselves using either NOVA classification or country-specific dietary guidelines. Based on the nutritional value, the most promoted food items on both online food delivery services and online grocery services also aligned with the discretionary food group classification of the Australian Dietary Guidelines.

#### Online food delivery services

Of the eleven studies that investigated online food delivery services, six studies [12, 28–30, 32, 34] reported the kinds of food and beverages that were promoted using online food marketing techniques. One [34] of the three [12, 28, 34] studies conducted in Brazil analysed 1593 food items on an online food delivery platform during 16 weeks of the COVID-19 pandemic, reported that the OFD platform most commonly promoted traditional meals and pasta, ultra-processed beverages and sandwiches (65%). These food groups were offered 20–25% of the time during the study period. The other two studies [12, 28] also reported a similar high proportion of ultra-processed beverages on offer in the apps (78%) compared with water (49%), natural juices or smoothies (27%). Ultra-processed ready-to-eat meals represented almost 70% of the food offered in the establishments’ menus, whilst traditional meals and vegetables represented just over 30% of the offering during the pandemic. Of the three other cross-sectional studies, one [30] reported that 69% (427/618) of food/beverage items offered on online food delivery services in Australia, the UK and the USA were classified as discretionary before and during the COVID-19 pandemic. Two other studies reported 73.3% (18,955/25,877) and 80.5% (11,139/13,841) of the food items as discretionary on New Zealand’s [32] and Australia’s [29] market-leading online food delivery platforms, respectively.

#### Online grocery services

Of the five studies that investigated online grocery services, only one study [9] classified 3473 foods and beverages marketed across 21 online grocery service retailers in the US into 13 mutually exclusive food groups based on the NOVA classification system. The study found that nearly two-thirds (64%) of marketed foods and beverages were ultra processed. Of the ultra-processed foods, 69% were excessive in sodium, 49% were excessive in free sugars, 40% were excessive in saturated fats, and 4% contained low- or no-calorie sweeteners. The top five marketed food and beverage categories reported were: candy, sweets and snacks (17.3%); fruit, vegetables and legumes (16.7%); bread, cereal and grains (10.8%); milk, yogurt and cheese (9.7%); and sugary drinks (6.9%).

#### Potential influence of the online marketing techniques on consumer’s food purchase behaviours

Only two studies [33, 41] assessed and reported a positive influence of brand perception (via good service quality, instant delivery and product quality) and price-centric promotions (via discount framing as absolute saving or percentage discount) on consumers’ purchasing, (re)purchasing intention and actual purchase behaviour towards foods on promotion on online food delivery services [33] and online grocery services [41]. Importantly, both these studies leveraged Cialdini’s principle of ‘scarcity’ and ‘authority’ to persuasively inform consumers’ purchase intentions and actual purchase behaviour.

#### Online food delivery services

One cross-sectional study conducted in Indonesia [42] surveyed 199 users of online food delivery services. The study reported critical ratio values (*t*-values; a priority index used to determine the order in which tasks should be processed, with values above 1.96 typically indicating statistical significance at the 0.05 level) for price-centric promotions, brand reputation, purchase intention and actual purchase. This study found that price-centric promotions (via discount framing as absolute saving or percentage discount) and brand reputation (trustworthy, reliable) had a positive effect on purchase intention to use the online food delivery service for their food purchase (CR value of 3.365 and 6.214, respectively). Further, the study found that food purchase intention had a positive effect on actual food purchase behaviour (CR value of 6.079).

#### Online grocery services

One cross-sectional study conducted in China [41] analysed survey data from 352 online grocery shoppers and showed that customer service (standardised regression coefficient (*β*) = 5 0.394, CR = 5.024, *p* < 0.001), price value (*β* = 0.435, CR = 5.728, *p* < 0.001), and instant delivery (*β* = 0.262, CR = 3.484, *p* < 0.001), significantly impacted consumers’ attitude towards online grocery services. However, attitude toward online grocery services did not positively impact continuous purchase intention (*β* = 0.154, CR = 1.352, *p* > 0.05) from online grocery services. But online attitude toward brick-and-mortar grocery retailers (*β* = 0.741, *p* < 0.001) had a significant influence on continuous purchase intention from online platforms of the brick-and-mortar grocery retailers.

## Discussion

### Key findings

This is the first scoping review to summarise evidence from business and health literature on the nature and potential influence of digital marketing techniques prevalent on three online food retail service types. We found that online food delivery services and online grocery services employ well-established digital marketing techniques to enhance and personalise creative marketing content. These were underpinned by four of Cialdini’s six principles of persuasion [25, 26], namely scarcity, liking, social proofing and authority. Whilst there is some evidence to suggest that these two online service types promote unhealthy food options, there is limited evidence examining the influence of the digital marketing techniques on consumers’ online food purchase behaviour. No studies examined marketing techniques on meal kit platforms, which reflects the market dominance of online food delivery and online grocery services and the overall scarcity of research on meal kits compared with these other platforms. This is a notable gap, given their increasing popularity and potential to shape cooking practices and dietary quality [42, 43].

Our review shows that online food retail settings have attracted interest across both business and public health disciplines, though with differing motivations. Predictably, studies published in business literature have largely focussed on how marketing techniques can enhance brand reputation, increase spending and build customer loyalty. This matters for public health because these techniques that deepen customer–brand relationships are also being harnessed to drive the promotion of highly processed, energy-dense foods [8, 44, 45]. For example, in our review we found studies published in the business literature across both online grocery services [38, 40] and online food delivery services [33, 36], utilised membership-based models, affiliate marketing and personalisation approaches to generate purposeful persuasive strategies to enhance branding and build customer relations. These techniques were also reported to promote unhealthy food products, as observed in studies in our review published in the public health literature [9, 30, 39].

Studies in our review revealed that both online food delivery and online grocery services adopt multiple marketing mix strategies, particularly price promotions and popularity cues purposively persuading consumers to select food product’s by highlighting their value for the money, convenience, tastiness and pleasure. For example, a few studies during the COVID-19 pandemic described how online food delivery services adapted their marketing strategies, emphasising themes like ‘combatting the pandemic’ [12, 30, 34]. The persuasive principles embedded within these marketing strategies reflect a growing body of evidence in business literature that demonstrates how persuasive marketing techniques are being designed in ways to shape consumer purchasing behaviours [46, 47]. The public health research examining the population health implications of these persuasive marketing strategies remains nascent [4]—highlighting a significant disciplinary gap. This underscores the urgency for public health researchers to actively engage with interdisciplinary evidence to better understand and counteract the influence of persuasive marketing strategies on health-related behaviours.

Only two studies [33, 41] (published in business journals) in our review examined the impact of marketing techniques within online food delivery services on consumer behaviour. Both reported a positive influence of price-centric promotions (as absolute savings or percentage discounts) on consumers’ food purchase intentions. Albeit limited, this reaffirms that consumers continue to seek value for money to influence their food purchasing intentions as observed in other settings, such as supermarket and grocery store environments [48]. This empirical evidence contributes to the growing body of research demonstrating the potential for digital marketing techniques to influence unhealthy food purchasing behaviours and, by extension, dietary risk. Such evidence is important for informing public health action to better address the influence of digital food marketing on population diets.

Online food retail settings present a significant opportunity to reach large consumer audiences and promote healthy foods, leveraging advanced algorithmic marketing techniques to influence consumer choices at scale [40, 49]. This underscores the need for further exploration into how online food retail platforms can be used to improve population diets. Future research could unpack whether and how existing in-store healthy food retail policies (for example, menu labelling [50]) can be adapted or implemented in the online food retail environment and what impact these may have on food purchase decisions [51, 52]. Identifying interventions and policies that balance public health benefits with commercial interests may help secure engagement from the food retail sector [53].

### Strengths and limitations

A key strength of our review is its interdisciplinary scope, integrating insights from both business and public health perspectives to provide a more comprehensive understanding of digital food marketing. This interdisciplinary approach strengthens our understanding of how commercial marketing practices intersect with public health concerns in the evolving digital food retail landscape. Another strength of this review is that most studies included were conducted after the COVID-19 pandemic and thus represent the current state of evidence on the online food environment. Our review has some limitations. Overall, there is a scarcity of relevant studies on the topic. Given the rapidly evolving online food retail platforms, the digital marketing techniques identified from the included studies may not be exhaustive. Future studies could continue tracking new, advanced, sophisticated forms of digital marketing techniques applied on online food retail platforms and ascertain their impact on online food choices. Another limitation is that our review did not consider marketing techniques used by online food retail settings on other platforms (such as YouTube), as this was beyond the scope of this review, which has also been shown to influence consumer food purchase behaviour [16].

## Conclusion

Our review demonstrates that online food delivery services and online grocery services utilise advanced marketing techniques to enhance and influence consumer experience. Whilst limited, our review also found some evidence suggesting that online food delivery services and online grocery services use these techniques to preferentially promote unhealthy food options. However, the evidence on the potential influence of the online marketing techniques on consumers’ food purchase behaviours, in real-world settings, is limited. From a public health perspective, this lack of empirical evidence is critical as it underscores the need for public health evidence to inform public health actions that improve the digital food environment to support population health. At the same time, there remains a continued need to better understand how online marketing techniques can be leveraged to support health, within the profit-driven nature of these platforms.

## Supplementary Information

Supplementary Material 1: Appendix A: PRISMA extension for Scoping Reviews (PRSIMA- ScR) checklist.

Supplementary Material 2: Appendix B: Search strategy.

Supplementary Material 3: Appendix C: Codebook for data synthesis.

## Data Availability

All data generated or analysed during this study are included in this published article [and its supplementary information files].

## Abbreviations

NCD: Non-communicable diseases
OFD: Online food delivery
UX: User Experience
SEM: Search engine marketing
SEA: Search engine advertising
SEO: Search engine optimisation
SMS: Short message service
UGS: Users to generate content
GPS: Global Positioning System
PROSPERO: The International prospective register of systematic reviews
PRISMA-ScR: Preferred Reporting Items for Systematic Reviews and Meta-Analyses extension for Scoping Reviews
PCC: Population, Concept, Context
SNAP: Supplemental Nutrition Assistance Program
