# Supplementary material for: Digital marketing techniques within online food retail platforms: a scoping review
Source: BMC Med. 2025 Dec 10;24:30. doi: 10.1186/s12916-025-04553-6 (PMC12801864; doi:10.1186/s12916-025-04553-6)
Supplement: Supplementary file 2 — Supplementary Material 2: Appendix B: Search strategy. [file 12916_2025_4553_MOESM2_ESM.docx]

Appendix B: Search Strategy

| Online food retail types (abstract) | AND | Intervention foci (text word) |
| --- | --- | --- |
| “Online food delivery” OR “online food delivery platform” OR “food delivery” OR “Online food delivery apps” OR "online food delivery service*” OR “OFD” OR “online meal delivery” OR “food service delivery” OR “online grocery” OR “meal kit*” OR “online food environment” OR “online supermarket” OR ”online restaurants” OR “direct delivery” OR “click and collect” |  | product OR place* OR profile OR portion* OR cost OR pricing OR price OR promotion* OR “food promot*” OR priming OR prompt* OR proximity OR discount* OR loyalty* OR voucher* OR nudge OR incentive* OR “brand” OR social media OR influencer* OR “paid advertise*” OR bonus* OR reward* OR coupon* OR token* OR rebate* OR refund* OR access* OR display OR layout OR activit* OR initiative* OR program* OR reformula* OR recipe* OR product* OR “email notification” OR “push notification” OR member* OR deal* OR combo* OR delivery fee OR “delivery time” OR photo OR image OR Marketing OR “Digital marketing” OR Advertising OR “food advertis*” OR tactic* OR “retail strategies” or “retail marketing” OR “online retail” OR “retail analytics” OR “targeted advertising” OR “targeted marketing” OR personalis* OR “web analytics” OR “website search engine optimisation” OR “SEO” OR “push notification*” OR “online checkout” OR “online-to-offline” OR “Online Systems” OR online OR internet OR Virtual OR Web OR “Web Based” OR digital OR “Internet-Based” OR Cyber OR “e-commerce” OR website OR app OR apps OR data mining |
