# Supplementary material for: Digital marketing techniques within online food retail platforms: a scoping review
Source: BMC Med. 2025 Dec 10;24:30. doi: 10.1186/s12916-025-04553-6 (PMC12801864; doi:10.1186/s12916-025-04553-6)
Supplement: Supplementary file 3 — Supplementary Material 3: Appendix C: Codebook for data synthesis. [file 12916_2025_4553_MOESM3_ESM.docx]

Appendix C: Codebook analysis

|  | **Digital Marketing techniques** | | | | | | | | | | **7Ps of the marketing mix strategies** | | | | | | | | **Cialdini’s six principles of persuasion** | | | | | |
| --- | --- | --- | --- | --- | --- | --- | --- | --- | --- | --- | --- | --- | --- | --- | --- | --- | --- | --- | --- | --- | --- | --- | --- | --- |
| **Author-Year (Reference)** | **User Experience Strategy (UX):** | **Search engine marketing (SEM)** | **Freemium to subscription-based** | **E-mail marketing (targeted email campaigns)** | **Mobile marketing (via SMS)** | **Pay-per-click advertising** | **Social media marketing** | **Affiliate marketing** | **Location based marketing** | **Retargeting strategy** | **Price** | **Promotion** | **Placement** | **Product Type** | **People** | **Process** | **Physical evidence** | **Others** | **Reciprocity** | **Commitment** | **Social Proof** | **Liking** | **Authority** | **Scarcity** |
| Online food delivery services | | | | | | | | | | | | | | | | | | | | | | | | |
| Horta PM., et al 2020 [38] |  |  |  |  |  |  |  |  |  |  | X | X |  | X |  |  |  | Photos |  |  |  | X |  | X |
| Horta PM., et al. 2020 [36] |  |  |  |  |  |  |  |  |  |  | X | X |  | X |  |  |  | Photos; Free delivery; Messages of healthiness, economy and tasty and pleasure. | X |  |  | X |  | X |
| Wang C., et al. 2021 [45] |  |  |  |  |  |  |  |  |  |  | X | X |  | X |  |  |  | Popularity cues, photos |  |  | X | X |  | X |
| Jia SS., et al. 2021 [39] |  |  |  |  |  |  | X | X |  |  | X | X |  |  |  |  |  | Photos, Health claims |  | X |  | X |  |  |
| Jitsoonthornchaikul M. 2022 [40]; |  |  | X |  |  |  |  |  |  |  | X | X | X | X | X | X | X |  |  |  |  |  | X | X |
| Mahawar N., et al. 2022[41] |  |  |  |  |  |  |  |  |  |  | X | X |  | X |  |  |  | Popularity cues, photos |  |  | X | X |  | X |
| Sari PN., et a. 2022 [44] |  |  |  |  |  |  |  | X |  |  | X |  |  |  |  |  |  | Brand reputation |  |  |  |  | X | X |
| Horta MP., et al. 2022 [37] |  |  |  |  |  |  |  |  |  |  | X | X |  |  |  |  |  | Photos; messages on healthiness, value for the money, tastiness, and pleasure |  |  |  | X |  | X |
| Rita P., et al. 2022 [43] |  | X |  |  |  |  |  |  |  |  |  | X |  |  |  |  |  | Customer reviews, customer satisfaction, word of mouth |  | X | X |  |  |  |
| Pandey A. and Wang, JX. 2023[42] |  |  |  |  |  |  |  | X |  |  | X | X | X | X |  |  |  | Brand reputation and consumer loyalty |  | X |  |  | X | X |
| Anil A., et al. 2023[32] |  |  |  |  | X |  |  |  |  |  |  | X |  |  |  |  |  | Increase customer engagement |  | X |  |  |  |  |
| Online Grocery Services | | | | | | | | | | | | | | | | | | | | | | | | |
| Banerjee T., and Banerjee A. 2016 [33] |  | X |  |  |  |  |  |  |  |  |  |  |  |  |  |  |  | Customer reviews, enhancing the visibility of their online grocery retail business |  |  | X |  | X |  |
| Headrick G., et al. 2021 [35] | X |  | X |  |  |  |  | X |  |  | X | X | X | X |  |  |  |  |  |  |  |  |  | X |
| Hallikainen H., et al., 2022 [34] |  | X |  |  |  |  |  |  |  |  | X | X | X |  |  |  |  | Consumer loyalty |  | X |  |  |  | X |
| Moran AJ., et al. 2022[20] | X |  | X |  |  |  |  | X |  |  | X | X | X | X |  |  |  | Product images, Popularity cues |  |  | X | X |  | X |
| Zhao Y., et al. 2023[46] |  |  |  |  |  |  |  |  |  |  | X | X | X | X | X | X |  | Instant delivery; Convenience | X |  |  |  | X | X |
